# Automated Particle Size Analysis of Supported Nanoparticle TEM Images Using a Pre-Trained SAM Model

**DOI:** 10.3390/nano15241886

**Published:** 2025-12-16

**Authors:** Xiukun Zhong, Guohong Liang, Lingbei Meng, Wei Xi, Lin Gu, Nana Tian, Yong Zhai, Yutong He, Yuqiong Huang, Fengmin Jin, Hong Gao

**Affiliations:** 1 School of Chemical Engineering and Technology, Tianjin University, Tianjin 300072, China; kun0428@tju.edu.cn (X.Z.); guohong.liang@tju.edu.cn (G.L.); xiwei@tju.edu.cn (W.X.); gulin_1213@tju.edu.cn (L.G.); nntiantju@hotmail.com (N.T.); zhaiyong@tju.edu.cn (Y.Z.); heyt@tju.edu.cn (Y.H.); huang_yq@tju.edu.cn (Y.H.); 2 School of Artificial Intelligence, The Chinese University of Hong Kong, Shenzhen 518172, China; lingbeimeng@link.cuhk.edu.cn

**Keywords:** supported nanoparticles, transmission electron microscopy, image segmentation, particle size analysis, deep learning, segment anything model

## Abstract

This study addresses the challenges associated with transmission electron microscopy (TEM) image analysis of supported nanoparticles, including low signal-to-noise ratio, poor contrast, and interference from complex substrate backgrounds. This study proposes an automated segmentation and particle size analysis method based on a large-scale deep learning model, namely segment anything model (SAM). Using Ru/TiO_2_ and related materials as representative systems, the pretrained SAM is employed for zero-shot segmentation of nanoparticles, which is further integrated with a custom image processing pipeline, including optical character recognition (OCR) module, morphological optimization, and connected component analysis to achieve high-precision particle size quantification. Experimental results demonstrate that the method retains robust performance under challenging imaging conditions, with a size estimation error between 3% and 5% and a per-image processing time under 1 min, significantly outperforming traditional manual annotation and threshold-based segmentation approaches. This framework provides an efficient and reliable analytical tool for morphological characterization and structure–performance correlation studies in supported nanocatalysts.

## 1. Introduction

Supported nanomaterials, which consist of active nanoparticles dispersed across a solid support matrix, have emerged as critical functional systems across a broad spectrum of technological applications, including new energy technologies (e.g., lithium-ion batteries and fuel cells) [1], electronic and information devices (e.g., semiconductors, sensors) [2], biomedicine (e.g., targeted drug delivery, bioimaging) [3], and environmental remediation (e.g., water purification, air pollution control) [4]. System performance is fundamentally driven by the high specific surface area, tunable interfacial chemistry, and engineered electronic structures of the supported nanoparticles, which collectively enable enhanced catalytic activity, selectivity, and functional responsiveness. For heterogeneous catalysis, in particular, the size and spatial distribution of nanoparticles critically determine the exposure of active sites, surface energy, and sintering behavior, which ultimately govern catalytic activity, selectivity, and long-term stability [5]. Therefore, to establish reliable structure–property relationships and to guide rational materials design, it is imperative to conduct high-precision characterization and statistical analysis of nanoparticle size and distribution in supported nanomaterial systems.

Common electron microscopy (EM) techniques, including scanning electron microscopy (SEM) and transmission electron microscopy (TEM), enable direct visualization of the size characteristics and spatial distribution of supported nanoparticles and have become indispensable tools in the analysis of supported nanomaterials [6]. In particular, TEM and high-angle annular dark-field scanning transmission electron microscopy (HAADF-STEM) offer high spatial resolution, exceptional sensitivity, and multifunctional analytical capabilities, making them essential for nanoscale characterization. Typically, EM images are analyzed manually by experienced researchers using image processing software such as ImageJ (version 1.54g) to obtain particle size and morphology data. However, the interpretation of such images often faces multiple challenges. Firstly, low signal-to-noise ratio and weak contrast cause small, highly dispersed nanoparticles to appear blurred against the background, leading to indistinct boundaries and potential omission of true particles [7]. Secondly, complex substrate backgrounds, including lattice fringes, structural defects, and amorphous boundaries, often overlap with nanoparticle signals, making edge detection difficult and boundary definitions ambiguous [8,9,10]. Thirdly, under high loading conditions or suboptimal synthesis control, nanoparticles tend to aggregate into irregular clusters with overlapping morphologies, resulting in further challenges in boundary separation and diverse particle shapes [11]. Consequently, even for skilled materials scientists, the process is time-consuming and susceptible to subjective bias. To achieve rapid and objective analysis, there is a pressing need for unbiased, efficient, and accurate automated methods for processing EM images.

In recent years, deep learning has demonstrated tremendous potential in the precise estimation of nanoparticle size parameters, enabling automatic learning and extraction of high-dimensional features directly from image data. For instance, Farley et al. proposed a U-Net-based segmentation approach for atomic force microscopy (AFM) nanostructure surface images, which exhibited superior noise resistance compared to traditional methods such as Otsu thresholding and local mean filtering [12]. Wu et al. [13] developed a particle characterization method that integrates in situ microscopic imaging with Mask R-CNN, enabling segmentation, classification, and size analysis of particles, and significantly improving detection accuracy for overlapping particles and multicomponent systems relative to conventional approaches. Bals and Epple constructed a deep learning-based automated workflow that employed UNet++ for nanoparticle segmentation in SEM/STEM images and combined AlexNet and ResNet34 for shape classification, thereby efficiently extracting both particle size and shape distribution information [14]. Fu and Aldrich [15] introduced a dense convolutional neural network (CNN) method that incorporates superpixel preprocessing (simple linear iterative clustering) with U-Net for online size analysis of ore particles on conveyor belts, demonstrating enhanced robustness under conditions of low illumination and particle overlap. However, the practical deployment of these deep learning models often requires extensive task-specific fine-tuning, including the preparation of large-scale training datasets, labor-intensive manual annotation, and time-consuming training on deep networks with millions of parameters. Therefore, the exploration of data-efficient representation and learning methods that do not rely on manual labeling has become a critical direction for advancing automated nanoparticle analysis.

Segment anything model (SAM) [16] is a foundational model for general-purpose image segmentation capable of segmenting novel and unseen objects with minimal prior training. By leveraging extensive pretraining on massive-scale datasets, SAM generalizes its segmentation capabilities far beyond the original training samples to a wide array of objects and scenes. Through deep learning of image feature distributions, SAM can adapt to diverse image types and application domains without the need for task-specific fine-tuning. It was applied in fields such as medical imaging, natural scene understanding, and geospatial remote sensing [17,18,19]. Owing to its unique ability to operate without annotated samples, SAM offers a new technical pathway for reducing annotation costs and accelerating segmentation workflows.

This study proposes an automated analysis framework based on SAM, designed to reduce the reliance on manual annotation in nanoparticle characterization. Operating under a zero-shot paradigm, the framework performs the segmentation of TEM images and integrates image processing with particle size statistical modules to enable quantitative analysis of nanoparticle size. The method was evaluated using Ru/TiO_2_ as a benchmark system and further extended to additional material systems including Cu/SiO_2_ and PdZn/TiO_2_. Its performance was compared against expert manual annotations. Experimental results demonstrate that, even without any task-specific training or labeled data, the segmentation accuracy of this framework is comparable to manual annotation, and it maintains excellent robustness under complex imaging conditions. Therefore, the proposed approach provides a rapid and efficient image analysis pathway for supported nanoparticle TEM data without the need for extensive labeling or retraining.

## 2. Materials and Methods

### 2.1. Method Overview

**Figure 1:**
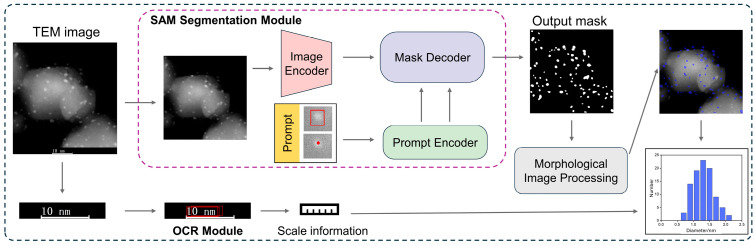
Framework for image recognition and statistical analysis of supported nanoparticles.

An analytical framework is proposed for supported nanoparticles (Figure 1), which comprises three core components: a nanoparticle segmentation module, a scale bar optical character recognition (OCR) module, and a particle size statistical module. First, the input image is processed by the SAM-based segmentation module, where the image encoder extracts features, the prompt encoder receives point or box prompts, and the mask decoder outputs the segmentation masks corresponding to individual nanoparticles [16]. To suppress small artifacts and isolated noise, the initial masks are refined using morphological opening operations and area-based threshold filtering. Next, the OCR module automatically detects and reads the scale bar embedded in the image to establish a pixel-to-nanometer conversion ratio. Finally, using the extracted scale information, the area and equivalent circular diameter of each segmented nanoparticle are converted from pixel units to physical dimensions (nm), and a particle size distribution histogram is generated.

#### 2.1.1. Image Segmentation Module

This study introduces the SAM, an advanced zero-shot segmentation model from the field of computer vision, as the backbone for segmenting TEM images. Pretrained on an exceptionally large dataset comprising 11 million images and over 1 billion masks, SAM has developed a powerful capacity for visual concept understanding and generalization. It enables high-quality segmentation with minimal manual effort through simple interactive prompts, even in the absence of labeled training data, thereby significantly reducing the labor and time costs traditionally associated with nanoparticle segmentation. The architecture of SAM consists of three key modules: an image encoder, a prompt encoder, and a mask decoder.

The image encoder is based on the vision transformer (ViT) architecture, which extracts global semantic features and multi-scale representations. Compared with conventional CNNs, Transformers are more effective at modeling long-range dependencies, which is particularly advantageous for distinguishing subtle contrasts between particles and background in noisy or low-contrast EM images [20]. Additionally, the rich prior knowledge accumulated from large-scale pretraining enables the ViT encoder to achieve excellent generalization performance, ensuring stable segmentation across diverse imaging scenarios.

To further enhance the model’s focus on relevant regions, the prompt encoder is designed to receive user-provided cues (e.g., point clicks or bounding boxes) to guide the segmentation process. In this study, an interactive web-based interface allows users to click on or outline nanoparticles within the image, which then serves as input to the SAM model (see Section 2.2 for details on the GUI and prompting workflow). This prompting mechanism enables the model to accurately distinguish nanoparticles from complex backgrounds, even in the presence of noise or low contrast, thereby substantially improving its adaptability to different imaging conditions.

The mask decoder uses the multi-scale features and prompts obtained from the previous modules to generate high-quality binary segmentation masks through progressive upsampling and feature fusion. A stepwise upsampling strategy is employed, in which the feature maps are gradually restored to the original resolution of the input image through multiple decoding stages.

Together, these components enable the SAM-based segmentation module to effectively and efficiently segment nanoparticles from TEM images. In contrast to conventional deep learning models such as U-Net, which require large amounts of annotated data and extensive retraining, SAM offers a lightweight and annotation-free alternative, requiring only simple user prompts while greatly enhancing the overall efficiency of nanoparticle image analysis.

#### 2.1.2. OCR and Particle Size Statistics Module

This study further introduces a particle size analysis module, whose primary function is to eliminate artifact interference from the segmentation masks and compute the physical diameter of each particle.

Firstly, the module checks whether the header of the input file contains scale-related metadata. For common microscope raw data formats such as DM3/DM4 and TIFF, we directly use their native metadata fields to obtain the scale information of the TEM image, thereby deriving a more accurate pixel-to-physical length conversion factor. For images whose metadata can be successfully parsed, the subsequent pipeline no longer relies on OCR, and thus does not introduce additional recognition errors.

When no valid scale information is available in the metadata, the system automatically switches to OCR mode. Specifically, the image is divided into small segments to detect elongated connected regions that indicate the presence of a scale bar, after which surrounding text is recognized to extract numerical scale values and corresponding physical units. To accomplish this, the study employs a lightweight Chinese–English OCR (CnOCR) toolkit based on the convolutional recurrent neural network (CRNN) deep learning model, which is well-suited for recognizing printed text and common unit symbols such as “nm/μm/Å”. Once the physical unit is identified, the length of the scale bar is measured in the pixel domain and, together with the extracted physical length, used to compute a pixel-to-physical conversion factor, which is subsequently applied to convert the diameter of each particle from pixel units to nanometers. In current implementation, the OCR module assumes a conventional TEM scale-bar layout: a single, high-contrast horizontal bar located near the image border (typically along the lower edge or close to a bottom corner), accompanied by a nearby numeric label in a standard, non-stylized font followed by a common unit abbreviation such as “nm/μm/Å”. Because the detection operates in the grayscale domain, the absolute color of the bar is not constrained, but sufficient intensity contrast with respect to the background and a roughly horizontal orientation are required for robust parsing; very low-contrast, strongly tilted, fragmented, or heavily stylized scale bars may therefore necessitate manual specification of the scale.

Following scale calibration, the particle size analysis module applies a multi-stage geometric analysis pipeline to refine the binary segmentation masks generated by the SAM model. A morphological opening operation is initially performed: in the erosion stage, small protrusions, point-like noise, and narrow “neck”-connected regions are removed; in the subsequent dilation stage, the main morphology of each particle is restored as accurately as possible. This process effectively eliminates isolated noise points and minor artifacts arising from image interference, retaining only those particle regions that are statistically meaningful.

Subsequently, a connected-component filtering step is carried out based on pixel area. Regions below a predefined lower threshold are classified as noise and removed, while an upper threshold may also be applied to eliminate unusually large, non-target structures such as shadows or stains. Both thresholds are defined in physical units and then converted to the pixel domain, ensuring consistent filtering criteria across images with varying magnifications.

Once each labeled region’s pixel-level data is obtained, the module calculates the projected area of each particle. Finally, based on the equivalent circle model, each particle is approximated as a circle with the same area as its projection, and its equivalent circular diameter is computed using the transformation Equation (1). By integrating this with the previously extracted scale factor from the OCR module, the pixel-based dimensions are ultimately converted into physical nanometer values, completing the particle size quantification process.

(1) $$D=\sqrt{\frac{4A}{\pi }},$$

where A represents the pixel area of the particle.

### 2.2. Web-Based Graphical User Interface and Prompting Workflow

To make the method accessible to materials researchers without programming experience, we implement an interactive, browser-based graphical user interface (GUI) that supports both interactive analysis of single images and batch processing of datasets acquired in the same experiment. The overall workflow consists of several key steps: scale calibration, interactive prompt input, size-based filtering, and result export.

#### 2.2.1. Scale Calibration and Single-Image Mode

The user first uploads TEM/HAADF-STEM images via the web interface. The GUI then calls the scale-extraction module described in Section 2.1.2 to automatically determine the scale: it first attempts to read the pixel size from the metadata header of raw microscopy formats such as DM/TIFF; if no valid metadata are detected, it automatically searches for the scale bar along the lower edge of the image, locates the bar, and applies OCR to recognize the adjacent numerical value and unit, from which a pixel–physical length conversion factor is inferred. The recognized scale is displayed in real time, and the user may manually correct it according to the scale annotation in the original image or directly enter the scale value, thereby eliminating errors caused by OCR failures or atypical scale-bar styles. Once the scale has been confirmed, the user proceeds to single-image segmentation and particle-size analysis.

#### 2.2.2. Interactive Prompts

In this work, SAM is guided in practice by a small number of interactive prompts that convey the regions of interest to be segmented. The GUI provides two of the most common and convenient prompt types: point prompts, where the user clicks near the centre of a particle to indicate a target that should be segmented, and box prompts, where the user draws a rectangular box that encloses the particles of interest. Prompt quality directly affects segmentation performance. For nanoparticles on relatively smooth backgrounds, with strong contrast against the support and clear boundaries, coarse boxes or sparse point prompts are often sufficient. For more complex regions with severe agglomeration or particle sizes comparable to the characteristic texture scale of the support, using tighter, particle-hugging boxes can substantially increase the probability that SAM correctly resolves individual particles and reduces missed detections or boundary mergers.

#### 2.2.3. Size Filtering

For images where no explicit ROI prompts are used, the lower-right panel of the GUI offers a particle-size filtering function. The user specifies minimum and maximum particle diameters in physical units, and the system automatically converts these thresholds into the pixel domain, removing regions smaller than the lower bound (noise or tiny fragments) and connected components that are clearly larger than the upper bound (non-target objects). This function is particularly useful for cleaning up numerous missegmented small fragments or background speckles in complex fields of view and, in combination with interactive prompts, helps focus the analysis on the particle-size range of interest.

#### 2.2.4. Batch Processing Mode

In many experiments, a large number of images are acquired under highly consistent conditions (for example, identical magnification, similar ROI locations, and a common scale). Building on the single-image mode, the GUI therefore provides a batch processing mode in which users first select one or several representative images, complete scale calibration, choose the prompting strategy, adjust the size-filtering thresholds, and run segmentation and particle-size analysis once in single-image mode. After confirming that this configuration yields satisfactory results on the representative images, they upload all images from the same experiment and start batch recognition with shared parameters. The system then automatically applies exactly the same SAM model and parameter configuration to the entire image set, performs segmentation and size analysis for each image without additional user interaction, and exports a compressed archive containing the mask overlays and the per-image statistical outputs. Throughout this process, the SAM model weights remain fixed and the parameter configuration is held constant within the batch, which avoids potential systematic bias arising from tuning parameters separately for different samples and considerably increases throughput for datasets from the same experiment.

#### 2.2.5. Export of Indexed Masks for Extended Morphological Analysis

In practical nanomaterials characterization, researchers are often interested in richer morphological information such as aspect ratio, circularity, and faceting or edge sharpness. To support such downstream custom analyses, the GUI, in addition to exporting particle-size histograms and overlay images, also exports indexed mask images: a binary mask image containing only the segmented particles, and a corresponding indexed label image in which each connected component is assigned a unique numeric ID. Users can then import these masks and index maps into ImageJ (version 1.54g), Python (version 3.11.13), or other analysis platforms and compute arbitrary shape descriptors (aspect ratio, morphological parameters, faceting indicators, etc.) based on the indexed particles without modifying the core pipeline.

### 2.3. Materials and Data Acquisition

The primary objective of this study is to evaluate the effectiveness of the prompt-guided zero-shot segmentation approach using the SAM for TEM and HAADF-STEM image analysis of supported nanoparticles, while simultaneously achieving automated particle identification and size quantification. To this end, this research prepared and acquired TEM and HAADF-STEM images of several representative supported nanoparticle systems, including Ru/TiO_2_, Cu/SiO_2_, and PdZn/TiO_2_. As a photocatalyst, Ru/TiO_2_ exhibits excellent performance in hydrogen production through water splitting. Surface modification of TiO_2_ nanoparticles with metal or non-metal dopants is a well-established strategy for enhancing electronic properties and improving hydrogen evolution efficiency [21]. Under ultraviolet, visible, or simulated solar illumination, these dopants play a critical role in promoting the separation of electron-hole pairs on the TiO_2_ surface. Furthermore, Ru/TiO_2_ shows promising potential in energy-related applications such as Fischer-Tropsch synthesis, which enables the conversion of coal-derived syngas into liquid fuels and valuable chemicals, contributing to the clean and efficient utilization of coal resources [22]. Conversely, Cu/SiO_2_ and PdZn/TiO_2_ represent widely used catalytic systems known for their high activity and selectivity. In particular, Cu/SiO_2_ has demonstrated strong potential in methane conversion, CO_2_ hydrogenation, and organic synthesis. Notably, in the CO_2_-to-methanol reduction reaction, both Cu/SiO_2_ and PdZn/TiO_2_ catalysts exhibit excellent selectivity and reactivity, effectively promoting CO_2_ conversion and fixation, thereby offering critical technological support for advancing global carbon neutrality initiatives.

#### 2.3.1. Reagents

TiO_2_ (P25) was purchased from Evonik Specialty Chemicals Co., Ltd. (Shanghai, China), and sulfuric acid (95–97%) was obtained from Jiangtian Chemical Co., Ltd. (Tianjin, China). All other reagents were supplied by Shanghai Aladdin Biochemical Technology Co., Ltd. (Shanghai, China), including RuCl_3_·xH_2_O (metal content ≥ 99.95%), NaBH_4_ (granular, 99.99%), CuCl_2_·2H_2_O (≥99%), SiO_2_ (≥99.99%), H_2_PtCl_6_·xH_2_O (≥99%), and ZnCl_2_ (≥99.95%).

#### 2.3.2. Synthesis of Materials

The Ru/TiO_2_ material was synthesized using a chemical reduction method [23]. Specifically, 0.5 g of P25 was dispersed in 50 mL of deionized water and stirred for 6 h to form a homogeneous suspension. Subsequently, 2 mL of RuCl_3_ solution (8.66 mg/mL) was added, followed by the dropwise addition of an excess of NaBH_4_ solution (6 mg/mL) until the suspension turned light gray in color. After an additional 2 h of stirring, the product was collected by centrifugation, washed multiple times with ethanol and deionized water, and dried overnight under vacuum, yielding Ru/TiO_2_ with a 3 wt.% loading. Finally, the dried sample was calcined in a tube furnace at 300 °C for 4 h, producing the final Ru/TiO_2_ powder.

The Cu/SiO_2_ and PdZn/TiO_2_ materials were synthesized using the same approach. For Cu/SiO_2_, 0.5 g of SiO_2_ was ultrasonically dispersed in 50 mL of deionized water and stirred for 6 h to obtain a uniform suspension. Subsequently, 43.7 mg of CuCl_2_·2H_2_O was added, followed by 30 min of stirring. Then, NaBH_4_ solution (6 mg/mL) was added dropwise at a rate of 1 mL/min until the suspension changed from light blue to colorless, accompanied by the formation of a light brown precipitate. An additional 20% excess of NaBH_4_ was added to ensure complete reduction of Cu^2+^. The reaction mixture was stirred for 2 h at room temperature, centrifuged, washed three times with ethanol and deionized water, and vacuum-dried at 80 °C for 12 h. The resulting powder was then calcined in a tube furnace under an air atmosphere, ramped at 5 °C/min to 300 °C, and held for 4 h, yielding Cu/SiO_2_ with an 8 wt.% loading.

For PdZn/TiO_2_, 0.5 g of P25 was dispersed in 50 mL of deionized water and stirred for 6 h, followed by the sequential addition of 1.5 mL of H_2_PtCl_6_ solution (8.66 mg/mL) and 0.5 mL of ZnCl_2_ solution (20 mg/mL) to achieve a Pd:Zn molar ratio of 1:1. After stirring for an additional 30 min, NaBH_4_ solution (6 mg/mL) was slowly added dropwise until the suspension turned from orange-yellow to dark gray, and then a 30% excess was added. The reaction was maintained at room temperature for 2 h, followed by centrifugation, washing, and vacuum drying at 80 °C for 12 h. The dried sample was then reduced in a 10% H_2_/Ar atmosphere using a temperature ramp of 2 °C/min up to 400 °C, held for 4 h, and allowed to cool naturally, resulting in PdZn/TiO_2_ with a 3 wt.% alloy loading.

#### 2.3.3. TEM Image Acquisition

The supported nanoparticles prepared in Section 2.3.2 were examined and characterized using JEOL TEM (JEOL Ltd., Akishima, Japan), specifically the JEM-F200 and JEM-ARM300F2 models. Prior to image acquisition, standard electron optics alignment procedures were performed, including sample height adjustment, beam centering, condenser aperture alignment, electron gun centering, voltage center adjustment, and correction of both condenser and objective lens astigmatism. Following these calibrations, TEM and HAADF-STEM images of the materials were acquired for subsequent analysis.

## 3. Results and Discussion

### 3.1. Evaluation Criteria

To objectively evaluate the performance of the zero-shot segmentation approach based on the pretrained SAM model, this study adopts four key metrics: overall relative error, distribution consistency, counting consistency, and small-particle sensitivity. These indicators are computed on a per-image basis and aggregated into an overall assessment by weighting according to the number of particles in each image.

#### 3.1.1. Data Configuration and Construction of Human “Consensus”

The modeled particle diameters can be expressed as follows:

(2) $$A\;=\;{\left\{a_{i}\right\}}_{i=1}^{n_{A}}$$

The manually annotated particle diameters by the *k*-th expert are given as follows:

(3) $$H_{k}\;={\;\left\{h_{k,j}\right\}}_{j=1}^{n_{H_{k}}}(k=1,2,3)$$

To avoid the complexity of one-to-one particle matching, alignment is performed in quantile space. A fixed quantile grid $p_{m}\in \;(0,\;1)$ is selected, and for each human annotator, the corresponding empirical quantile function $Q_{H_{k}}(p)$ is computed. A consensus quantile function is then defined to represent the aggregate annotation across annotators.

(4) $$Q_{H}\left(p\right)=\mathrm{median}(Q_{H_{1}\left(p\right)},Q_{H_{2}\left(p\right)},Q_{H_{3}\left(p\right)})$$

where $Q_{H}\left(p\right)$ represents the quantile function derived from the software output. All subsequent comparisons are performed over the paired quantile grid points defined by the set $\left\{Q_{A}(p_{m}),Q_{H}(p_{m})\right\}$.

#### 3.1.2. Core Metrics and Computations

(1) Quantile Mean Absolute Percentage Error (Primary Metric)

The overall relative error (${\mathrm{MAPE}}_{Q}$, where lower is better) is a distribution-level metric based on quantile alignment, designed to avoid one-to-one particle matching. It remains robust when the sample sizes are unequal or when outliers are present in either set. Furthermore, its percentage form facilitates cross-scale comparisons, with the following equations:

(5) $${\mathrm{MAPE}}_{Q}\;=\;\frac{100\%}{M}\sum_{m=1}^{M}\frac{\left|Q_{A}\left(p_{m}\right)-(p_{m})\right|}{Q_{H}(p_{m})}$$

(2) Bland-Altman Consistency Analysis (Bias and Limits of Agreement (*LoA*))

Let the difference be denoted as $d_{m}\;={\;Q}_{A}\left(p_{m}\right)-Q_{H}\left(p_{m}\right)$, the overall relative error is defined as follows:

(6) $$\bar{d}\;=\;\frac{1}{M}\sum_{m=1}^{M}d_{m}$$

(7) $$s_{d}=\sqrt{\frac{1}{M-1}}\sum_{m=1}^{M}{\left(d_{m}-\bar{d}\right)}^{2}$$

(8) $$\mathrm{LoA}=(\bar{d}+1.96s_{d})-(\bar{d}-1.96s_{d})$$

(9) $$\rho_{\mathrm{LoA}}=\frac{1.96s_{d}}{\bar{Q_{H}}}\times 100\%,\;\bar{Q_{H}}=\frac{1}{M}\sum_{m=1}^{M}Q_{H}(p_{m})$$

where $\bar{d}$ represents the systematic bias; *LoA* represents the width of the 95% agreement interval—both are preferred to be as small as possible; and $\rho_{\mathrm{LoA}}$ represents the relative half-width of the agreement interval, serving as a scale-invariant metric that more appropriately captures consistency across different imaging fields and particle size magnitudes.

(3) Relative First-Order Wasserstein Distance (Distributional Distance ($W_{1}^{\mathrm{rel}}$))

To quantify the difference between two distributions, the following metric is expressed as follows:

(10) $$W_{1}^{\mathrm{rel}}\;=\;\frac{1}{\bar{Q_{H}}}\cdot \frac{1}{M}\sum_{m=1}^{M}\left|Q_{A}\left(p_{m}\right)-Q_{H}\left(p_{m}\right)\right|$$

(4) Count Consistency (Detection Rate Proxy)

Using the median particle count from human annotations as the reference, the following equation can be obtained:

(11) $$n_{\mathrm{ref}}\;=\;\mathrm{median}(n_{H_{1}},n_{H_{2}},n_{H_{3}}),\;\epsilon_{n}\;=\;\frac{n_{A}-n_{\mathrm{ref}}}{n_{\mathrm{ref}}}$$

where $\epsilon_{n}$ represents the overall rate of over-detection or under-detection, providing an intuitive quantification of the segmentation result’s deviation at the level of object count.

(5) Small-Particle Ratio Difference (Low-Contrast Sensitivity ($|∆\phi \left(t\right)|$))

Let a threshold *t* = 3 nm. The following metric is defined:

(12) $$\phi_{A}\left(t\right)\;=\;\frac{1}{n_{A}}\sum_{i}1(a_{i}\leq t)$$

(13) $$\phi_{H}\left(t\right)=\frac{1}{n_{H}}\sum_{j}1(h_{j}\leq t)$$

(14) $$∆\phi \left(t\right)=\phi_{A}\left(t\right)-\phi_{H}\left(t\right)$$

A smaller value of $|∆\phi \left(t\right)|$ indicates that the sensitivity to small particles is more closely aligned with human annotators.

### 3.2. Analysis and Discussion of Recognition Results

**Figure 2:**
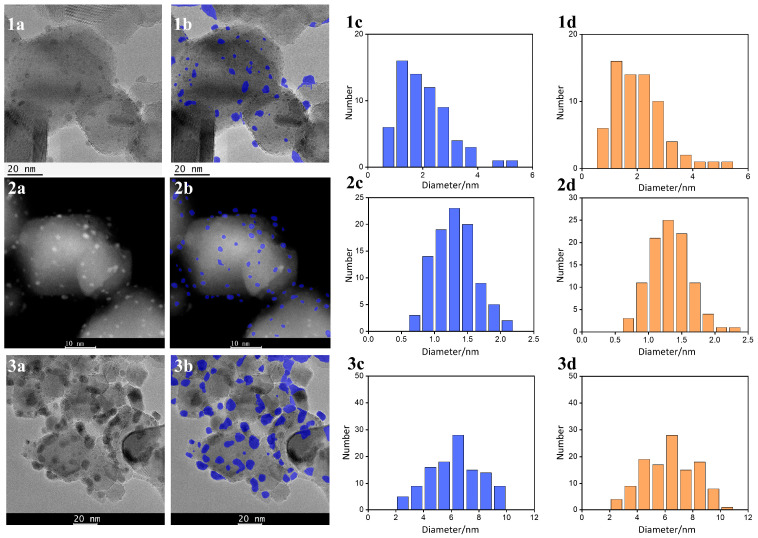
Comparison of TEM images and particle size statistical analyses of Ru/TiO_2_ samples under different regions or conditions. For each region (rows 1–3), panels (**1a**,**2a**,**3a**) show the original TEM images; (**1b**,**2b**,**3b**) show the SAM-based automatically segmented images (blue masks highlighting the nanoparticles); (**1c**,**2c**,**3c**) show the particle size distributions obtained by the proposed automatic method (blue histograms); and (**1d**,**2d**,**3d**) show the corresponding distributions obtained from manual annotation (orange histograms).

By combining the zero-shot segmentation capabilities of the pretrained SAM model with a custom-developed pipeline comprising scale bar OCR, morphological optimization, and connected component analysis, high-precision automatic particle identification and size distribution analysis were performed on TEM and HAADF-STEM images, using Ru/TiO_2_ and Cu/SiO_2_ as representative examples. To quantitatively evaluate the effectiveness of the proposed method, this study selected TEM/HAADF-STEM images of the same field of view, and compared the automated results with those from manual measurements. Manual annotations were performed in ImageJ (version 1.54g), including scale calibration and particle size analysis, and were independently completed by three annotators with TEM expertise. In Figure 2, the particle size distributions obtained by the proposed method are highly consistent with the results derived from manual, particle-by-particle annotations, providing initial validation of the model’s robustness and accuracy, particularly under conditions of complex background interference and low-contrast nanoparticles.

Furthermore, based on the particle size data obtained from the proposed method and the three human annotators, a multi-dimensional evaluation was conducted. The analysis includes ${\mathrm{MAPE}}_{Q}$, $\left|\bar{d}\right|$, $\rho_{\mathrm{LoA}},$ $W_{1}^{\mathrm{rel}}$, $\epsilon_{n}$, and $|∆\phi \left(t\right)|$. The results of this comprehensive assessment are summarized in Table 1.

The evaluation results show that ${\mathrm{MAPE}}_{Q}$ ranges from 0.78% to 1.87%, while the $W_{1}^{\mathrm{rel}}$ lies between 0.80% and 1.79%. In the Bland-Altman analysis, the $\bar{d}$ is no greater than 0.006 nm, and the $\rho_{\mathrm{LoA}}$ remains below 5%. These results demonstrate that, even under challenging conditions such as low contrast, complex support textures, and large particle size variation, the proposed zero-shot segmentation method, combined with scale bar OCR, morphological optimization, and connected component analysis, is capable of maintaining the overall deviation from manual measurements within approximately 2%, without introducing significant systematic bias. This level of accuracy meets the requirements for high-throughput particle size and dispersion statistics.

A further breakdown by imaging modality and particle size regimes indicates that the proposed method maintains strong sensitivity and consistency in the small-particle range. Using threshold-based evaluation *t* = 3 nm, the corresponding $|∆\phi \left(t\right)|$ values for the three cases are 3.730%, 0%, and 0.56% (Figure 2(1a–3a)), all within 4%, indicating high agreement with human annotations for particles smaller than 3 nm. However, under more complex conditions, such as strong agglomeration, irregular morphologies, or blurred boundary signals variability in performance is observed. In particular, when particle sizes approach the lattice periodicity of the support or when particles are partially embedded in the support surface, segmentation can be adversely affected by support texture interference or tangential/connected particles, leading to degradation in $\rho_{\mathrm{LoA}}$ and $\epsilon_{n}$. Figure 2(1a) exemplifies such scenarios $\rho_{\mathrm{LoA}}$ = 4.48%, $\epsilon_{n}$ = −7.04%), where the $|∆\phi \left(t\right)|$ is slightly elevated due to missed detection of extremely small particles, open contours, or occlusion within agglomerated clusters. By comparison, Figure 2(2a) (with all particle sizes ≤ 3 nm) and 2(3a) (dominated by larger particles) illustrates more stable performance in terms of distributional error and small-particle detection rates, though localized fluctuations may still occur in the presence of significant agglomeration or non-spherical particles. Overall, HAADF-STEM imaging, with its superior small-particle contrast and narrower intensity profiles $\rho_{\mathrm{LoA}}$, appears more favorable for enhancing consistency. In contrast, TEM images are more susceptible to mild under-detection and boundary ambiguity under conditions of low contrast, lattice interference, or embedded interfaces. However, the primary distribution-level metrics remain within a 1–2% error margin, supporting the method’s robustness for high-throughput analysis even in challenging imaging environments.

Compared with conventional segmentation networks that require supervised training on task-specific datasets, the “zero-shot segmentation approach based on the pretrained SAM model” proposed in this study exhibits three significant advantages. Firstly, it is annotation-free, training-free, and plug-and-play. Without the need for labeled data or model retraining, it can directly produce particle size distributions that are highly consistent with those obtained by multiple human annotators (${\mathrm{MAPE}}_{Q}$ ≈ 1%, $\rho_{\mathrm{LoA}}$ ≤ 4.5%) significantly reducing the cost and complexity associated with model development and maintenance. Secondly, it demonstrates strong robustness across diverse imaging scenarios. The three evaluated images cover challenging conditions including low-contrast TEM, high-density small-particle distributions, and mixed mid-to-large particle regimes. Under all these conditions, the method maintains consistent distributional performance ($W_{1}^{\mathrm{rel}}$ ≤ 1.8%) indicating that the pretrained visual priors of SAM generalize well to complex backgrounds and varying imaging conditions. Thirdly, the pipeline is closed-loop and produces calibrated, interpretable results. It incorporates scale bar OCR to automatically convert pixel measurements to physical dimensions, and uses morphological operations and connected component analysis for reliable post-processing and standardized measurements (e.g., area-equivalent diameters). This ensures unified statistical outputs, facilitating cross-sample comparisons and large-scale batch processing.

### 3.3. Processing Efficiency and Throughput

Under standardized hardware and I/O conditions (Intel i9-14900K + NVIDIA A100), the proposed end-to-end pipeline—comprising “image loading → zero-shot segmentation → quantitative analysis → result export”—achieves a per-image processing time of 19–33 s for original 4 k × 4 k TEM/HAADF-STEM images (median ≈ 25 s). More specifically, image preprocessing (file I/O, scale parsing, basic normalisation) takes approximately 1–2 s per image; the zero-shot SAM forward pass is the dominant computational cost at roughly 15–25 s per image; and the post-processing steps (morphological filtering, connected-component analysis, feature extraction, particle-size histogram computation, and generation of overlay images) add a further ~3–6 s per image. In contrast, manual particle-by-particle measurement for the same type of image typically requires 30–120 min/image. In the newly introduced batch-processing mode, preprocessing for a given experiment only involves parameter selection (scale, thresholds, size filters, etc.) on the first (or a few) representative images and can be regarded as a one-time overhead for the entire batch. Thereafter, model inference and post-processing run independently on each image with the above runtimes. Using the median runtime of 25 s per image, a batch of 100 images requires approximately 2500 s (≈42 min) on a single A100 GPU, while processing 10^3^ images takes about 2.5 × 10^4^ s (≈6.9 h). Based on this, the estimated total time to process 100 images can be reduced from over 60 h to under 1 h (approximately 42 min at 25 s/image), enabling a transition from minute-scale to second-scale analysis without the need for additional training or parameter tuning. This represents a multi-order-of-magnitude acceleration while substantially reducing both the manual labor burden and result variability associated with repetitive human annotation. With the same time budget (e.g., 1 h), the pipeline could theoretically process up to 144 images/h, a throughput unattainable by manual methods. Importantly, as shown in the consistency analysis, this speed-up does not compromise accuracy; for challenging cases involving very small particles or agglomerated regions, lightweight post-processing (e.g., increased prompt density, cluster separation, or local contrast enhancement) can further reduce the rate of manual review and correction, all while maintaining second-level latency. Overall, the pipeline strikes a practical balance between end-to-end latency, batch-scale throughput, and engineering usability, making it well-suited for routine high-volume characterization and rapid statistical analysis across sample batches.

### 3.4. Reproducibility and Analytical Drift

With a fixed SAM checkpoint and analysis parameter configuration, the workflow is fully deterministic: there is no online model training, no random data augmentation, and all post-processing steps are based on explicit, rule-driven operations. Once the user has completed parameter tuning on the first sample, the same configuration is applied to all images within that batch. In batch-processing mode, the pipeline automatically performs segmentation and particle size quantification for every image in the folder, and exports both the overlay images and the corresponding particle size distributions as a compressed archive. Re-running the same batch under identical settings produces identical outputs, thereby ensuring good reproducibility and statistical consistency even when processing hundreds or thousands of images.

From the model perspective, the toolchain always uses a fixed pretrained SAM checkpoint and does not perform any form of online updating or data-driven fine-tuning during routine use; as a result, the model itself does not exhibit any spontaneous drift over time. Potential differences in the results mainly arise from two sources: (i) deliberate changes to analysis parameters made by the user, and (ii) running the same dataset under different software or dependency versions. To mitigate such cumulative analytical drift in long-term structure–function studies, we recommend keeping the software and model versions fixed within the same data batch and storing a documented parameter preset for each data type. When updating the software version or changing the model checkpoint, one should first perform a comparative evaluation on a small set of representative images against the previous version, and only merge new analysis results with existing datasets once the observed differences are deemed acceptable.

### 3.5. Extended Sample Results and Discussion

**Figure 3:**
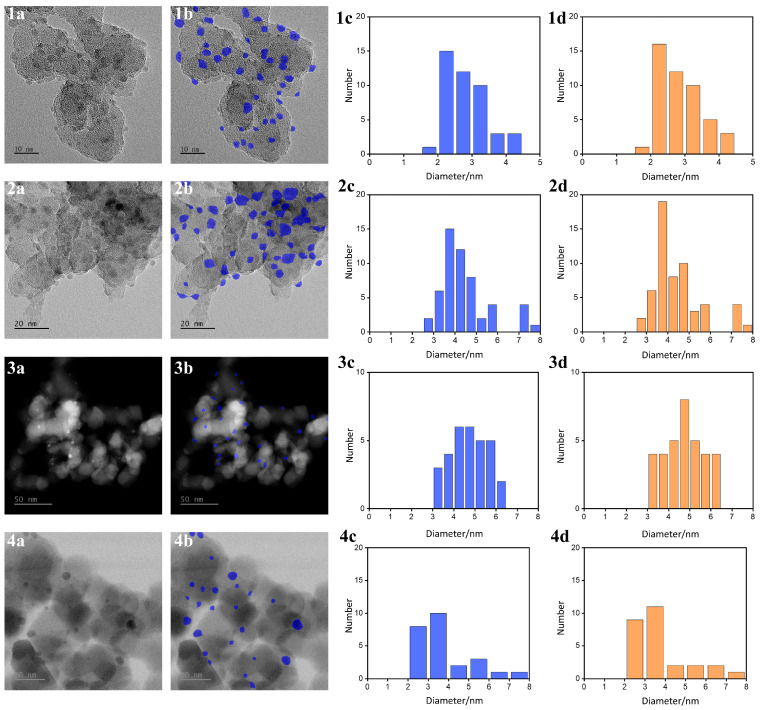
Comparison of TEM images and particle size statistical analyses of Cu/SiO_2_ (rows 1 and 2) and PdZn/TiO_2_ (rows 3 and 4) samples under different regions or conditions. For each region, panels (**1a**,**2a**,**3a**,**4a**) show the original TEM images; (**1b**,**2b**,**3b**,**4b**) show the SAM-based automatically segmented images (blue masks highlighting the nanoparticles); (**1c**,**2c**,**3c**,**4c**) show the particle size distributions obtained by the proposed automatic method (blue histograms); and (**1d**,**2d**,**3d**,**4d**) show the corresponding distributions obtained from manual annotation (orange histograms).

Particle size distribution analysis was conducted on TEM images of Cu/SiO_2_ samples (Figure 3(1a–d,2a–d)) and PdZn/TiO_2_ samples (Figure 3(3a–d,4a–d)) under different regions and conditions. The results were compared between the automatic segmentation based on the SAM and manual annotations. In Figure 3, each sample group includes the original TEM image, the corresponding SAM-based segmentation output, the automatically computed particle size distribution, and the averaged results from manual annotations. In this study, the zero-shot SAM-based segmentation method demonstrated high accuracy and robustness for particle size analysis in TEM images. According to the evaluation results in Table 2, the ${\mathrm{MAPE}}_{Q}$ remained below 3% for all samples, with Figure 3(1a) achieving ${\mathrm{MAPE}}_{Q}$ of 0.98%, indicating a high level of agreement between automated and manual size statistics. As sample complexity increased, ${\mathrm{MAPE}}_{Q}$ slightly rose but remained within an acceptable range, peaking at 2.46%. Additionally, the $\left|\bar{d}\right|$ values were close to zero, suggesting strong agreement with human estimations; notably, bias values were as low as 0.0239 nm and 0.0360 nm in Figure 3(1a,2a), respectively. In terms of *LoA*, all samples showed widths below 12.3%, and even the most complex case (Figure 3(4a)) remained within a reasonable range, reflecting the model’s stability and adaptability across diverse sample conditions. The $W_{1}^{\mathrm{rel}}$ also confirmed a high degree of consistency between the segmented and manually labeled size distributions, with all values below 2.5%, further validating the model’s accuracy. However, as sample complexity increased, the $\epsilon_{n}$ became more pronounced. Figure 3(1a,2a) depicts minor under-detection, Figure 3(4a) presents a larger detection rate error of −7.41%, indicating a noticeable underestimation of particle count. Regarding small particle detection, the differences in small-particle ratios in Figure 3(2a,3a) were minimal (3.70% and 0%, respectively), suggesting good sensitivity in these cases. Conversely, larger differences were observed in Figure 3(1a) (4.06%) and Figure 3(4a) (5.04%), indicating a decrease in small-particle detection performance as sample complexity increased. In summary, although certain under-detection and proportion discrepancies were observed under more challenging conditions, the SAM-based segmentation method still provides effective support for particle size analysis, demonstrating strong application potential.

### 3.6. Applicability and Limitations of Zero-Shot Pretrained SAM Segmentation Across Imaging Scenarios

Combining the results for Ru/TiO_2_ (Figure 2, Table 1) with those from the extended Cu/SiO_2_ and PdZn/TiO_2_ samples (Figure 3, Table 2), the applicability and limitations of the pretrained SAM in a zero-shot setting can be summarized in a scenario-based manner along three dimensions: imaging conditions, structural features, and domain shifts.

First, under typical TEM/HAADF-STEM conditions with weak contrast, relatively high noise levels, and moderately complex support textures, zero-shot SAM segmentation combined with scale-bar OCR, morphological refinement, and connected-component analysis yields highly stable distribution-level errors across different samples. The particle-size quantile errors (${\mathrm{MAPE}}_{Q}$, $W_{1}^{\mathrm{rel}}$) are generally controlled within 1–2%, the Bland–Altman bias $\left|\bar{d}\right|$ is close to zero, and the relative half-width of the 95% limits of agreement $\rho_{\mathrm{LoA}}$ is mostly below 5%. This indicates that, within the range of low-dose and weak-contrast acquisition and typical noise spectra, the pretrained SAM is fairly tolerant to domain shifts in contrast mechanism and noise level.

Second, in fields of view dominated by sub-3 nm nanoparticles with high number density and sizes approaching the resolution limit (e.g., Figure 2(2a) and Figure 3(1a)), the method maintains good sensitivity to weak-contrast small particles under suitable imaging conditions. The values of ${\mathrm{MAPE}}_{Q}$ remain below 2%, $W_{1}^{\mathrm{rel}}$ is around 2%, and the small-particle proportion difference $|∆\phi \left(t\right)|$ is typically within 4%. This shows that, within the pixel sizes and contrast range covered in this study, zero-shot SAM still provides robust detection sensitivity and distributional agreement for near-resolution nanoparticles.

Third, the most challenging cases for the model arise from polycrystalline and strongly textured supports and irregular particle morphologies. In Ru/TiO_2_ regions with pronounced lattice fringes, step defects, or rough textures (e.g., Figure 2(2a) and Figure 3(4a)), ${\mathrm{MAPE}}_{Q}$, and $W_{1}^{\mathrm{rel}}$ remain below 3%, so the overall distribution-level error is still within an acceptable range. However, $\rho_{\mathrm{LoA}}$ can widen to 10–12.3%, while the count error $\epsilon_{n}$ and the small-particle proportion difference $|∆\phi \left(t\right)|$ can reach about −7% and ~5%, respectively. Qualitatively, these degradations are mainly driven by two typical failure modes: (i) in regions with strong agglomeration or particles partially sintered into or embedded in the support, local boundary contrast is strongly reduced, and SAM tends to merge multiple nanoparticles into a single mask, leading to under-counting and overestimation of the equivalent diameter; and (ii) when the characteristic length scales of support features (lattice fringes, step edges, rough textures) are comparable to the nanoparticle size, the model may misclassify some support features as particles or produce blurred interfaces, thereby weakening its ability to distinguish nanoparticles from a complex substrate.

### 3.7. Future Extensions: Boundary-Guided, Morphology-Aware, and Multimodal Segmentation

In this work, boundary-guided, morphology-aware, and multimodal segmentation strategies are discussed as potential extensions, rather than components of the current baseline implementation. Conceptually, a boundary-guided refinement module would primarily target regions where SAM tends to produce merged masks in densely agglomerated clusters. Starting from the coarse SAM masks, local edge information (e.g., gradient magnitude, Canny/Sobel edges) could be used to detect internal “saddle points” within large connected components, and a lightweight, edge-aware watershed or distance-transform-based splitting could be applied only in these ambiguous regions. Such a module is expected to reduce under-segmentation and improve particle counting and size estimates in compact clusters, while adding only modest local post-processing cost and leaving the SAM backbone unchanged.

A morphology-aware component could build on simple shape descriptors derived from connected components (e.g., circularity, aspect ratio, convexity, solidity). These descriptors may be used (i) to filter out obvious artefacts and background fragments that are inconsistent with plausible nanoparticle shapes, and (ii) in suitable cases, to optionally regularize extremely irregular masks by fitting simple geometric primitives (e.g., ellipses). This would help stabilize size statistics in the presence of complex support textures or imaging artefacts, while keeping the pipeline fully deterministic and computationally light.

Finally, multimodal extensions could be realized at the post-processing layer without modifying SAM itself. For example, when multiple contrast modes (BF/DF/HAADF) are available for the same region, SAM could be run independently on each channel and the resulting masks fused via logical rules or confidence-based schemes. Alternatively, acquisition metadata (dose, voltage, support type) could be used to select among a small number of pre-defined pre-/post-processing parameter presets. Both approaches are expected to improve robustness under challenging imaging conditions by exploiting complementary information, while preserving the zero-shot nature, inference speed, and model generality of the current framework.

## 4. Conclusions

This study presents a deep learning-based approach that integrates the pretrained foundation model SAM with a custom-developed image analysis pipeline to enable automated segmentation and particle size quantification of supported nanomaterials in EM images. Using Ru/TiO_2_ and related samples as representative cases, the proposed method achieves rapid identification and high-precision extraction of nanoparticle size data from complex imaging environments, offering robust data support for constructing structure–property relationships and demonstrating strong potential as an intelligent assistant tool for experimental analysis. Compared to traditional commercial particle sizing software, the method maintains excellent segmentation robustness under challenging conditions such as low signal-to-noise ratio, poor contrast, complex substrate textures, and dense agglomeration, significantly reducing manual annotation workload and simplifying the image analysis workflow. It also exhibits superior adaptability and stability across diverse nanomaterial imaging scenarios. However, the study also reveals performance fluctuations in accuracy when dealing with severely agglomerated, irregularly shaped particles, or regions with ambiguous boundaries, particularly when particle sizes approach the lattice periodicity of the support or when particles are partially embedded in the support matrix. Future improvements may involve incorporating more diverse training data (e.g., low-dose imaging, low-contrast images, and polycrystalline structures) along with adopting structure-aware segmentation strategies (e.g., boundary-guided methods, morphology-aware embedding, or multi-modal fusion) to further enhance recognition capabilities and statistical precision for complex particle morphologies. Overall, the proposed method not only improves the efficiency and consistency of particle size analysis but also provides a scalable, stable, and intelligent toolchain for quantitatively exploring structure-function relationships in heterogeneous catalysis, laying a data-driven foundation for advancing mechanistic understanding of supported nanomaterials’ catalytic behavior.

## Figures and Tables

**Table 1:** Summary of consistency between automated and manual particle size statistics from representative Ru/TiO_2_ samples.

Sample Name	${\mathrm{MAPE}}_{Q}$ (%)	$|\bar{d}|\;$ (mm)	$\rho_{\mathrm{LoA}}$ (%)	$W_{1}^{\mathrm{rel}}$ (%)	$\epsilon_{n}$ (%)	|∆ϕ(t)| (%)
Figure 2(1a)	1.87	0.0047	4.48	1.79	−7.04	3.73
Figure 2(2a)	0.78	0.0030	2.09	0.80	−5.00	0
Figure 2(3a)	1.12	0.0052	3.10	1.12	−6.56	0.56

**Table 2:** Summary of consistency between automated and manual particle size statistics from Cu/SiO_2_ and PdZn/TiO_2_ samples.

Sample Name	${\mathrm{MAPE}}_{Q}$ (%)	$|\bar{d}|\;$ (mm)	$\rho_{\mathrm{LoA}}$ (%)	$W_{1}^{\mathrm{rel}}$ (%)	$\epsilon_{n}$ (%)	|∆ϕ(t)| (%)
Figure 3(1a)	0.98	0.0239	4.8	1.03	−6.38	4.06
Figure 3(2a)	1.26	0.0360	6.19	1.46	−5.26	3.70
Figure 3(3a)	1.50	0.0396	8.82	1.46	−8.82	0
Figure 3(4a)	2.46	0.0431	12.3	2.47	−7.41	5.04

## Data Availability

The code is available at https://github.com/Xiukun-Z/nanoSAM (accessed on 8 December 2025).
